# Eliminating bovine tuberculosis in cattle and badgers: insight from a dynamic model

**DOI:** 10.1098/rspb.2015.0374

**Published:** 2015-06-07

**Authors:** Ellen Brooks-Pollock, James L. N. Wood

**Affiliations:** 1 Disease Dynamics Unit, Department of Veterinary Medicine, University of Cambridge, Madingley Road, Cambridge CB3 0ES, UK; 2 School of Social and Community Medicine, University of Bristol, Oakfield House, Oakfield Grove, Bristol BS8 2BN, UK

**Keywords:** bovine tuberculosis, type reproduction numbers, transmission dynamics, disease control

## Abstract

Bovine tuberculosis (BTB) is a multi-species infection that commonly affects cattle and badgers in Great Britain. Despite years of study, the impact of badgers on BTB incidence in cattle is poorly understood. Using a two-host transmission model of BTB in cattle and badgers, we find that published data and parameter estimates are most consistent with a system at the threshold of control. The most consistent explanation for data obtained from cattle and badger populations includes within-host reproduction numbers close to 1 and between-host reproduction numbers of approximately 0.05. In terms of controlling infection in cattle, reducing cattle-to-cattle transmission is essential. In some regions, even large reductions in badger prevalence can have a modest impact on cattle infection and a multi-stranded approach is necessary that also targets badger-to-cattle transmission directly. The new perspective highlighted by this two-host approach provides insight into the control of BTB in Great Britain.

## 1. Introduction

Bovine tuberculosis (BTB) is a multi-species infection that has a serious impact on the cattle industry in Great Britain, as well as elsewhere. Despite extensive control measures in cattle, the disease in cattle remains uncontrolled and now costs the UK government approximately £100 million per year [1].

An investigation by the Ministry of Agriculture, Fisheries and Food into BTB outbreaks in cattle first found infection in the European badger (*Meles meles*) in 1971 [2,3]. Since then, a growing body of evidence has demonstrated a close linkage between the local disease in cattle and badgers. Molecular typing has demonstrated that the cattle and badgers in the same geographical area are usually infected with identical strains, although it is not possible to infer the direction or frequency of transmission from existing data [4,5]. The Randomised Badger Control Trial (RBCT) found a significant reduction in new cattle herd incidents associated with pro-active badger culling, indicating badger-to-cattle transmission [6]. Using RBCT data, Donnelly & Hone [7] estimated that badgers contributed to up to 52% of herd-level infections, but later estimated that only 5.7% were directly caused by badgers, when onward cattle-to-cattle transmission was excluded [8]. During the RBCT, infection in badgers also increased when cattle testing was reduced due to the foot and mouth epidemic in 2001, suggesting that cattle-to-badger transmission is also an important aspect of the system [9]. Despite this growing body of evidence, the disease is still frequently discussed as either a disease of cattle, or a disease of wildlife. Without a clear conceptual framework that describes the quantitative dynamics of this multi-host disease in different ecosystems, our understanding of and ability to manage the complex disease ecology will be unnecessarily impaired.

If BTB is truly a two-host infection in high incidence areas in Great Britain, then it is essential to consider both cattle and badger populations dynamically to quantify the long-term effects of control strategies in either host. Simple estimates of the reproduction number of BTB in badgers range from 1.03 to 1.35 [10–13] and estimates of the reproduction number in cattle range from 1.01 to 4.9 [14–17]. It is unclear what the relationship is between species or how reducing one value will affect the other. Controlling multi-host infections requires a different approach to single-host pathogens [18,19]. In a multi-host setting, amplification and feedback between hosts plays a critical role in the persistence of infection, and control focused on one host has a nonlinear impact on the whole system.

In Great Britain, a cattle test-and-slaughter surveillance scheme forms the basis of BTB control, although many modifications and additions have been introduced or proposed over time [1]. More recent cattle controls include pre- and post-movement testing to reduce transmission between farms, follow-up testing of persistently infected herds with the gamma-interferon blood test and more frequent routine testing, either annually or even every six months [1]. Risk-based surveillance and trading schemes and cattle vaccination are under active consideration. Badger controls, including culling and more recently vaccination, have been trialled in various forms since 1973, mainly in high incidence areas [6]. Biosecurity measures such as fencing and building modification have been used to reduce cattle–badger contact [20]. Predicting the impact of present and future controls requires an improved understanding of the dynamics of this two-host system.

This paper considers situations in the high incidence areas in Great Britain where the infection cycles between cattle and badgers. We describe and explore a two-host model of BTB transmission between cattle and badgers. Using established estimates of reproduction numbers together with estimates from RBCT data, we are able to identify plausible regions of parameter space. Our analysis provides a better understanding of the system and its nonlinearities and captures the direct and indirect impact of control strategies, identifying the long-term effects of targeting control at either a single-host or at inter-host contact.

## 2. Methods and results

### (a) Model specification

The model we used is a deterministic, Suscepible-Infected epidemic model with two hosts, cattle and badgers. We chose a two-state model based on previous models [14–16], as TB natural history justifies the exclusion of a ‘recovered’ state and because the focus of this analysis is equilibrium dynamics which are not affected by the inclusion of latent or occult periods. Other complexities, such as super-excretors, intermittent shedding or age-specific differences are not addressed here. Cattle and badgers are either susceptible to infection (proportions *S*_C_ and *S*_B_) or infected and infectious (*I*_C_ and *I*_B_). The transmission rates within and between hosts are denoted by *β*_CC_, *β*_CB_, *β*_BC_ and *β*_BB_. Infected cattle are removed at rate *γ*_C_, which represents removal via the test-and-slaughter scheme. Background turnover occurs in the cattle and badger populations at rates *μ*_C_ and *μ*_B_, respectively. Births and deaths are balanced to keep population sizes constant so *μ*_C_(*S*_C_ + *I*_C_) + *γ*_C_*I*_C_ represents cattle births and *μ*_B_(*S*_B_ + *I*_B_) represents badger births. Mortality in BTB-infected badgers was shown not to be significantly greater than in uninfected badgers. The increased mortality in super-excreting badgers [21] is not captured here.

The model equations are:2.1
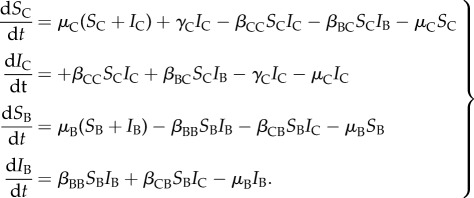


### (b) Number of secondary cases and type reproduction numbers

The type reproduction number, as defined by Roberts & Heesterbeek [18,19], is the sum of the average number of secondary cases produced by direct within-host transmission and the average number of secondary cases produced via indirect transmission. To derive expressions for the type reproduction numbers in cattle and badgers, we define the next generation matrix (NGM) as
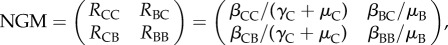
where *R*_CC_ is the number of secondary cases in cattle due directly to cattle and *R*_BC_, *R*_CB_ and *R*_BB_ are the number of secondary cases in cattle due to badger-to-cattle transmission, in badgers due to cattle-to-badger transmission and in badgers due to badgers, respectively. The reproduction numbers in the NGM describe the number of directly transmitted secondary cases, rather than the total impact of an infected animal caused by amplification in the other population. For instance, the average infected cattle will infect *R*_CC_ other cattle directly and *R*_CB_ badgers. These badgers will generate *R*_CB_*R*_BB_ more infected badgers, which in turn will generate *R*_CB_(*R*_BB_)^2^ infected badgers, then *R*_CB_(*R*_BB_)^3^ and so on. The outbreak in badgers caused by this initially infected cow will each generate *R*_BC_ infected cattle, therefore, the total number of secondary cases in cattle generated by an average cow will be2.2
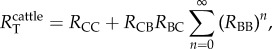
which is defined by Roberts & Heesterbeek [18,19] as the type reproduction number. A similar argument for the number of secondary cases in badgers caused by an average infected badger yields2.3
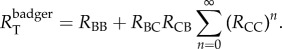


Although the notation is different, these are equivalent to the expressions derived by Roberts & Heesterbeek [18,19]. The geometric sum on the right-hand side of equations (2.1) and (2.2) has the potential to amplify the type reproduction number for the other host and if unbounded, can be a cause of continual spillover infection. We note that only the product *R*_BC_*R*_CB_ impacts the type reproduction numbers, therefore, reducing either one will affect transmission in both populations.

### (c) Infection prevalence at equilibrium

The equilibrium levels of infection in each population, 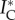 and 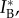 are affected by both the within-host transmission rates and the transmission rates of the other host. For instance, an increase in cattle-to-badger transmission will directly increase badger prevalence and indirectly increase cattle prevalence. Solving the model equations at equilibrium yields the coupled equations for the prevalence (see the electronic supplementary material for the derivations):2.4
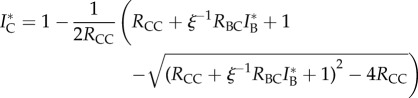
and2.5
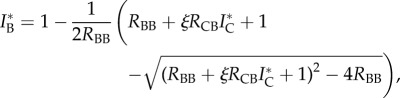
where *ξ* = (*γ*_C_ + *μ*_C_)/*μ*_B_ is the ratio of removal rates in cattle to badgers (see the electronic supplementary material for derivations). Equations (2.4) and (2.5) can be solved by iteration or other means. Within-host transmission and removal exhibit threshold behaviour where after a critical point disease is sustained within the population, whereas spillover from the other host has a monotonic impact with no critical points.

### (d) Mechanisms for sustained transmission in cattle and badgers

**Figure 1.**
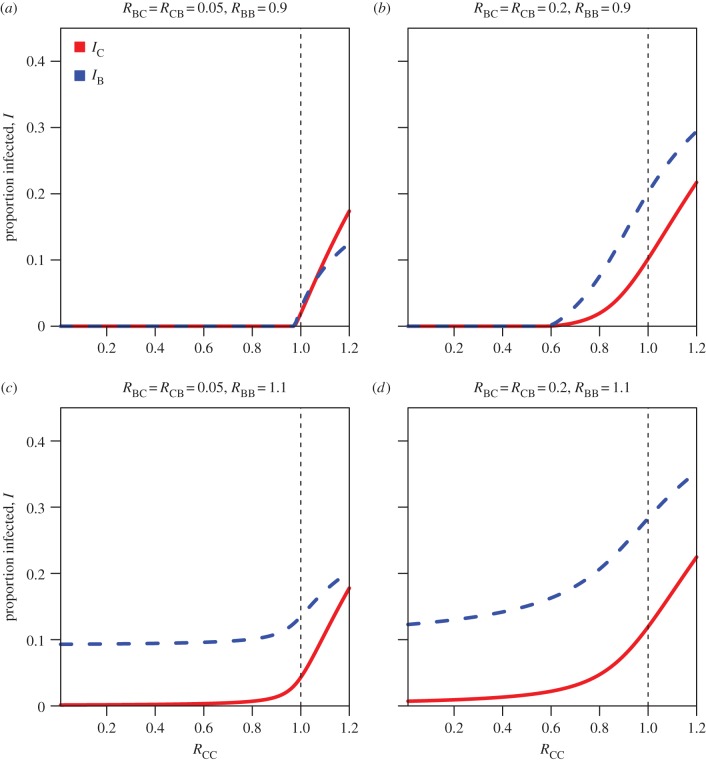
Four scenarios for bovine tuberculosis transmission between cattle and badgers in Great Britain. The horizontal axis *R*_CC_ is the number of secondary cases in cattle due directly to cattle and the vertical axis is the proportion of infected cattle (red) and infected badgers (blue) at equilibrium. *R*_BC_, *R*_CB_ and *R*_BB_ are the number of secondary cases in cattle due to badgers, in badgers due to cattle and in badgers due to badgers (see main text for details). The four scenarios are: (*a*) low inter-species transmission and unsustained transmission in badgers; (*b*) intermediate inter-species transmission and unsustained transmission in badgers; (*c*) low inter-species transmission and sustained transmission in badgers; and (*d*) intermediate inter-species transmission and sustained transmission in badgers.

Even in this simple model there are multiple qualitatively different scenarios for sustaining infection in the cattle population (figure 1*a–d* and the electronic supplementary material figures). Table 1 contains a list of all parameters with references. We characterize four scenarios based on the magnitude of the cattle-to-cattle reproduction number *R*_CC_ as the parameter with the greatest impact on cattle infection. The impact of other reproduction numbers on equilibrium levels of infection is explored in the supplementary information. The four scenarios, one corresponding to each panel in figure 1, are:

(i) low inter-host transmission (*R*_BC_ = *R*_CB_ = 0.05) and unsustained transmission in badgers (*R*_BB_ < 1);
(ii) intermediate inter-host transmission (*R*_BC_ = *R*_CB_ = 0.2) and unsustained transmission in badgers (*R*_BB_ < 1);
(iii) low inter-host transmission (*R*_BC_ = *R*_CB_ = 0.05) and sustained transmission in badgers (*R*_BB_ > 1);
(iv) intermediate inter-host transmission (*R*_BC_ = *R*_CB_ = 0.2) and sustained transmission in badgers (*R*_BB_ > 1).

Figure 1, which shows the equilibrium prevalence in cattle and badgers, was computed numerically using equations (2.4) and (2.5). The code to generate all figures is available as the electronic supplementary material download. In scenario (i), infection is driven solely by cattle and the badger population experiences spillover infection. Eradication in the cattle population can be achieved with *R*_CC_ close to, but less than 1 (*R*_CC_ < 0.975; figure 1). In scenario (ii), the cattle population still drives transmission but feedback and amplification in the badger population means that the eradication threshold in cattle is reduced further to *R*_CC_ < 0.6. In terms of infection in cattle, scenario (iii) appears similar to scenario (ii), with control achievable via cattle measures alone. However in scenario (iii), the cattle population will still experience sporadic outbreaks via spillover from the endemically infected badger population. Cattle control measures will have least impact on cattle infection in scenario (iv) where there is endemic infection in the badger population and high transmission from badgers to cattle.

**Table 1.** The notation, interpretation, values and references for parameters used in the model.

parameter	interpretation	values	references
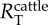	the type reproduction number in cattle	1.01–4.9	[14–17]
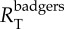	the type reproduction number in badgers	1.03–1.3	[10–13]
*R*_CC_	the number of secondary cases in cattle directly caused by cattle	0.94 (0.74, 0.99)	calculated from [7,8] and [14–17]
*R*_BC_	the number of secondary cases in cattle caused by badgers	0.049 (0, 0.12)	calculated from [7,8]
*R*_CB_	the number of secondary cases in badgers caused by cattle	0.05–0.2	estimated using [9]
*R*_BB_	the number of secondary cases in badgers caused directly by badgers	0.1–0.99	estimated using [9,10–13]
*μ*_C_	the background mortality rate of cattle	0.1 (1/year)	[16]
*μ*_B_	the background mortality rate of badgers	0.2 (1/year)	[21]
*γ*_C_	the removal rate of infected cattle	0.6–0.8 (1/year)	[15,16]

### (e) The magnitude of transmission within and between cattle and badgers

In a two-host system, reproduction numbers estimated in a single host are type reproduction numbers if transmission from the other host was not explicitly accounted for. Published estimates for 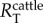 in the presence of the controls in GB are 1.1 [14], 1.5–4.9, where transmission scaled with herd size [15,17] and 1.01 [16]. Estimates of 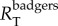 are 1.1–1.2 [10], 1.025–1.229 [11] and 1.03–1.35 [13]. The latter estimates were noted to increase with the period of observation. As discussed above, type reproduction numbers are unbounded if the within-host reproduction number in the alternate host is greater than 1. This is because a single infection can lead to sustained transmission in the other host that will be a continual source of reintroduction. Therefore, a type reproduction number in badgers that increases with observation time would be consistent with *R*_CC_ > 1. However, if both type reproduction numbers 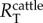 and 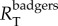 are greater than 1 but bounded, it follows then that the geometric sums 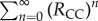 and 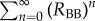 must also be bounded, and that *R*_CC_ and *R*_BB_ must be less than 1. If such values operate in a similar geographical area, it implies that neither population can sustain infection in isolation and both are reliant on feedback loops and amplification in the other host. In this case, we derive a simple relationship between the type reproduction number in cattle and the type reproduction number in badgers:2.6
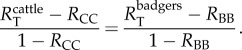


This reveals that the host with the greater type reproduction number (with both greater than one) has the lower host-specific reproduction number (when both are less than one). So, if the type reproduction number in cattle were larger than the type reproduction number in badgers then the optimal strategy would be to target badgers and vice versa. This counterintuitive result is due to amplification in the other host.

### (f) Interpreting cattle incidence data

**Figure 2.**
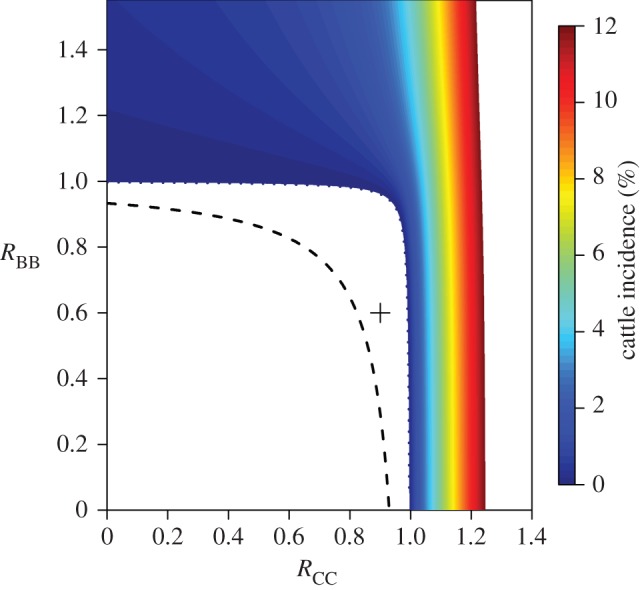
Incidence in cattle as a function of *R*_CC_ and *R*_BB_ with *R*_BC_ = *R*_CB_ = 0.05. The coloured areas represent incidence between 0.02% and 12%. White areas indicate combinations of values outside this incidence range. The blue dotted line marks the boundary of incidence greater than 0.02%. The black dashed line indicates the same boundary for *R*_BC_ = *R*_CB_ = 0.25 and the cross marks the point *R*_CC_ = 0.9 and *R*_BB_ = 0.6 (see text for discussion).

From national testing data, average incidence in herds with at least one reactor is approximately 2%, although there is wide variation between herds; 8% of positive herds have an incidence greater than 5%. Figure 2 shows the range of values of *R*_CC_ and *R*_BB_ consistent with incidence rates between 0.02% and 12%, assuming an inter-host mixing rate of *R*_CB_ = *R*_BC_ = 0.05. Using cattle incidence data alone, it is not possible to distinguish between cattle- or badger-driven transmission in low incidence herds. There is a trade-off between cattle and badger transmission such that low values of *R*_CC_ and high values of *R*_BB_ can produce the same incidence in cattle as high values of *R*_CC_ and low values of *R*_BB_. However, cattle herds with high incidence are unlikely to be sustained through transmission from badgers alone as *R*_CC_ must be greater than 1.04 to achieve an incidence of 4% per annum. The black dashed line in figure 2 shows the criteria for persistent infection (incidence rate greater than 0.02%) for an increased inter-host mixing rate of *R*_CB_ = *R*_BC_ = 0.25. For high levels of badger–cattle–badger transmission, infection can persist in the cattle population without sustained transmission in either population.

To illustrate equation (2.6), the cross in figure 2 marks where *R*_CC_ = 0.9 and *R*_BB_ = 0.6. At this point, a type reproduction number in badgers of 2.0 would imply a type reproduction number in cattle of 1.55, potentially falsely identifying the population with the greater transmission.

### (g) Using results from the Randomised Badger Control Trial

Using RBCT data, Donnelly & Nouvellet [8] estimated 52% (95% CI: 9.1–100%) of cattle infections were due to badgers (DN_1_), but that only 5.7% (95% CI: 0.09–25%) were as a result of direct badger-to-cattle transmission (DN_2_). The Donnelly and Nouvellet model did not include cattle-to-badger transmission (i.e. *R*_CB_ = 0), under the assumption that infection of wildlife from cattle was negligible owing to regular cattle testing. Applying this assumption to our model, the NGM is of the form
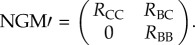


The number of cattle infections caused by badgers including onward transmission in cattle is 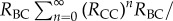 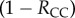. Therefore,
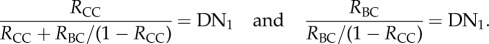
Using the values of DN_1_ = 52% (9.1–100%) and DN_2_ = 5.7% (95% CI: 0.09–25%) leads to *R*_CC_ = 0.94 (0.75–0.99) and *R*_BC_ = 0.049 (0–0.12), calculated using the two-dimensional posterior estimates from Donnelly & Nouvellet [8]. From figure 2, to achieve a cattle incidence of greater than 1% when *R*_CC_ = 0.94, *R*_BB_ must be greater than 0.96. These results rule out scenario (i), where the disease dynamics are driven solely by the cattle population and suggest that the most likely scenarios in RBCT areas (chosen for their high incidence of cattle TB) are scenarios (ii) or (iii).

**Figure 3.**
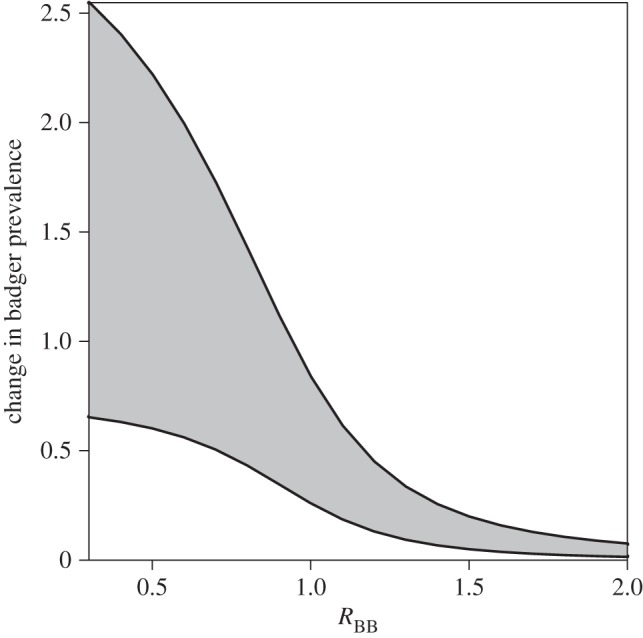
The increase in badger prevalence when cattle removal rate is reduced for values of *R*_BB_. The lower line represents the change in badger prevalence when cattle removal decreases from 0.8 to 0.5 years^−1^ and the upper line represents the change in badger prevalence when the cattle removal rate decreases from 0.8 to 0.2 years^−1^.

In order to identify the likely magnitude of *R*_BB_, we use the reported twofold increase in badger prevalence associated with a reduction in cattle testing during the 2001 foot and mouth disease (FMD) epidemic [9]. By simulating a decrease in cattle removal rate *γ*_C_, we find that if *R*_BB_ > 1.5, cattle testing has almost no impact on infection prevalence in badgers (figure 3). Although cattle testing was reduced during the 2001 FMD epidemic, it was not stopped completely. Using national test data, we estimate that *γ*_C_ dropped by approximately 40% (see the electronic supplementary material for details of this estimate). Investigating a range of removal rates from 15% to 50%, we find that the observed change in badger prevalence can be reproduced by combinations of *R*_CB_ and *R*_BB_ and that the most likely values are for *R*_BB_ < 1 and *R*_CB_ < 0.2.

Therefore, we conclude that parameters most consistent with the RBCT and testing data are *R*_CC_ = 0.94, *R*_BC_ = 0.05, *R*_CB_ < 0.2 and *R*_BB_ ≤ 1, and in high incidence herds, *R*_CC_ > 1. These parameters are most consistent with scenarios (ii) and (iii). It is worth noting that both scenarios (ii) and (iii) result in a higher prevalence in badgers than cattle, owing to the high removal rate of cattle.

### (h) Controlling infection in cattle

**Figure 4.**
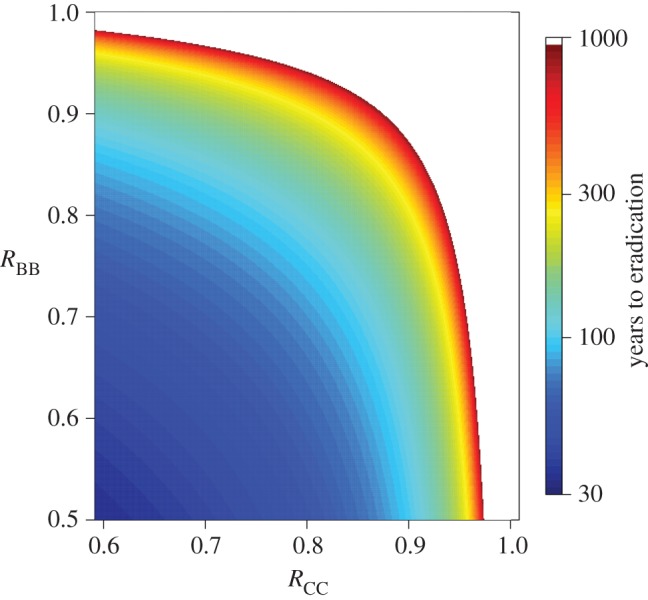
The time to achieve eradication in cattle (defined as less than five reactors per 10 000 cattle) as a function of *R*_CC_ and *R*_BB_ with *R*_BC_ = *R*_CB_ > 0.05. The white region indicates areas where eradication is impossible.

**Figure 5.**
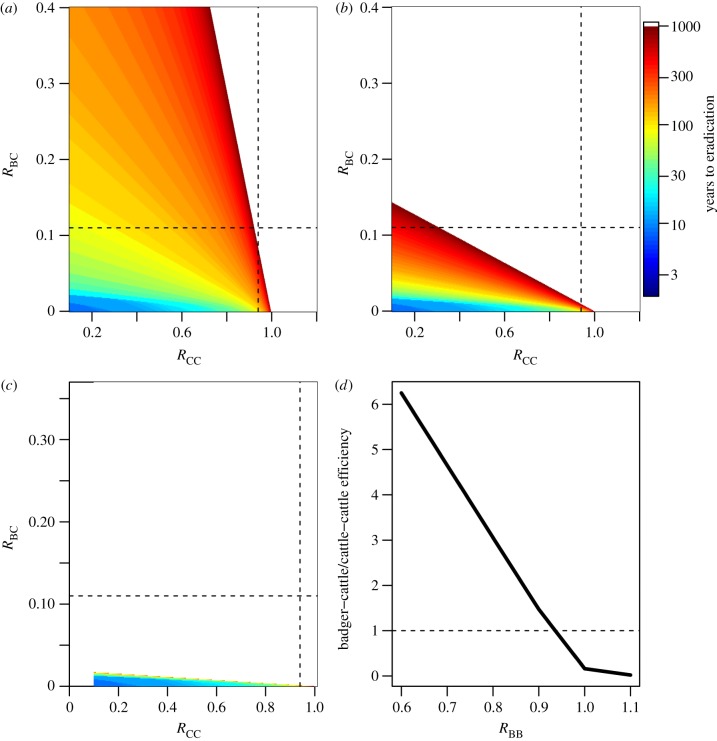
The time to achieve eradication in cattle (defined as less than five reactors per 10 000 cattle) as a function of (*a*) *R*_CC_ and *R*_BC_ with *R*_BB_ = 0.9 and *R*_CB_ = 0.1; (*b*) *R*_CC_ and *R*_BC_ with *R*_BB_ = 1.0 and *R*_CB_ = 0.1; and (*c*) *R*_CC_ and *R*_BC_ with *R*_BB_ = 1.1 and *R*_CB_ = 0.1. In (*a*–*c*), the vertical line denotes *R*_CC_ = 0.94 and the horizontal line *R*_BC_ = 0.11. The white region indicates areas where eradication is impossible. (*d*) The relative efficiency of reducing badger-to-cattle transmission compared with cattle-to-cattle transmission once cattle transmission is under control *R*_CC_ < 1.

Using the parameter estimates derived in the previous sections, figures 4 and 5 illustrate the targets that need to be achieved in order to bring cattle disease under control. Each figure was produced by simulating the model in equations (2.1). The colour represents the time necessary to bring infection rates in cattle to less than five reactors per 10 000 cattle tested (consistent with current low incidence areas) from a starting condition of *I*_C_ (*R*_CC_ = 1.05, *R*_BC_ = *R*_CB_ = 0.05, *R*_BB_ = 1.05) and *I*_B_ (*R*_CC_ = 1.05, *R*_BC_ = *R*_CB_ = 0.05, *R*_BB_ = 1.05) (dark blue is less than 10 years, dark red is over 1000 years and white is disease persistence where eradication is not possible). Figures 4 and 5*a*–*c* are not symmetric because the target for control is eradication in the cattle population, irrespective of prevalence in badgers.

On the right of figures 4 and 5*a*–*c*, when *R*_CC_ > 1, control in cattle cannot be achieved by any form of badger control measures. In these high incidence cattle herds, additional cattle controls must be introduced to control infection. Eradication becomes feasible as *R*_CC_ is reduced via cattle control measures. Conversely, at the top of each panel, when badger transmission is high, infection in cattle is sustainable even for *R*_CC_ < 1.

Figure 4 shows the concave relationship between *R*_CC_ and *R*_BB_. The shape of the relationship indicates that as transmission becomes sustainable in the cattle population alone, the impact of controlling badger transmission diminishes. For instance, considering the RBCT estimate of *R*_CC_ = 0.94, we note that even large reductions in *R*_BB_ may have a limited impact on eradication in cattle. At this level of cattle transmission, reducing *R*_BB_ from 0.6 to 0.0 (by 100%) only accelerates eradication time in cattle by 30% (from 92 to 64 years). In a different situation where cattle transmission is almost completely controlled but badger transmission is relatively high (top left hand corner of figure 4), controlling badger transmission is the only option for cattle eradication. If we assume that *R*_BB_ ≈ 0.9 then the most efficient method for achieving control would be to target both cattle and badger transmission simultaneously.

The impact of controlling badger-to-cattle transmission versus cattle-to-cattle transmission is considered in figure 5*a*–*d*. Both sources of infection have a direct impact on cattle eradication. Figure 5*a* shows the trade-off between cattle-to-cattle transmission versus badger-to-cattle transmission for *R*_BB_ ≈ 0.9, i.e. when transmission in the badger population is not sustainable. The magnitude of cattle-to-cattle transmission ultimately defines whether control is possible, however, with sufficient transmission from badgers, infection in cattle is sustainable when *R*_CC_ < 1. Comparing figure 5*a* with figure 5*b*,*c* illustrates the impact of badger-to-badger transmission. As badger-to-badger transmission increases, infection becomes sustainable in the cattle population, even in the absence of cattle-to-cattle transmission.

The gradient of the boundary between eradication and persistence dictates the relative efficiency of targeting badger-to-cattle transmission compared to cattle-to-cattle transmission once cattle transmission is under control, i.e. for *R*_CC_ < 1 (figure 5*d*). The steeper the gradient, the smaller the role of badgers in cattle incidence. In figure 5*d*, the vertical axis shows the number of badger-to-cattle infections that would have to be prevented to be equivalent to preventing a single cattle-to-cattle infection for achieving eradication in cattle. Thus, when the ratio is 1 it is equally effective to target cattle-to-cattle transmission as badger-to-cattle transmission. As can be seen in figure 5*a*–*c*, the eradication gradient ultimately depends on the average number of secondary cases produced by an average infectious badger, *R*_BB_. For values of *R*_BB_ > 0.93, it is more effective to target badger-to-cattle transmission, but for *R*_BB_ < 0.93 it is always more effective to target cattle-to-cattle transmission (figure 5*d*).

## 3. Discussion

Bovine tuberculosis is an infectious disease of cattle, badgers and other mammals that poses a serious threat to the livestock industry in Great Britain. Despite extensive cattle measures, control has proved elusive. Many changes to cattle testing have been introduced in the recent past, including an expansion of annual cattle testing and surveillance around detected herds in low incidence areas. Both badger vaccination and culling are being trialled in select areas. The interaction between controls targeted at badgers and cattle could produce complex dynamics without a straightforward interpretation.

In this paper, we propose that a reason for the difficulty in control and the seemingly variable impact of control measures is that the system is close to eradication. In this situation, infection in cattle and badgers depends critically on amplification and feedback from the other host species. Using published data together with a two-host dynamic model for cattle and badgers, we demonstrated that cattle prevalence may be relatively insensitive to badger controls but that close to the eradication threshold, our ability to control infection in cattle through cattle measures is highly sensitive to small changes in transmission from badgers. These results highlight the complex dynamics of eradication in Great Britain and illustrate the necessity of considering both host species as dynamical populations. The model results provide insight into control of the epidemic in the medium to long term.

The type reproduction number, introduced by Roberts & Heesterbeek [18,19], illustrates the difficulty for control in multi-host systems. For a disease with more than one host, infection can propagate in a secondary host, amplifying the reproduction number in the primary host. In this framework, the type reproduction number differs from the basic reproduction number for directly or vector transmitted infections as its impact may occur over an extended period of time—potentially longer than the primary host's lifetime.

The model we used was intentionally simple to allow for analytic traction. However, there are a number of caveats to our analysis. First, we emphasize that we did not attempt to capture many of the complexities of the BTB epidemic in Great Britain. There is much inter-farm variation in BTB risk due to cattle-level, farm-level and regional factors. For instance, a large cattle herd with several hundred cattle is more likely to be able to sustain infection through cattle-to-cattle transmission alone and badger controls will have smaller impact. Conversely, smaller herds in areas with high badger prevalence are likely to experience a greater benefit of a reduction in external transmission pressure. Second, using the model we are not able to comment on the feasibility of controls, reducing cattle transmission may be more or less achievable than reducing badger transmission by a similar amount. In this analysis, we have not attempted to capture the perturbation effect associated with culling badgers. Other analyses have focused on modelling controls realistically and future work could combine realistic control implementations in a dynamic two-host model. However, we find that reducing badger-to-cattle transmission is likely to be more effective than reducing prevalence in badgers alone. This may have particular implications for badger vaccination programmes, depending on the local incidence of badger infection.

Using relatively limited data, we were able to draw broad conclusions about the relationship between badger and cattle controls in Great Britain. Increased cattle controls, such as the universal annual testing now introduced in high incidence and ‘edge’ areas, are predicted to benefit all herds and result in a decrease in average breakdown size. Increased badger controls, resulting in a reduction in badger-to-cattle transmission, are likely to be most beneficial to low risk herds in high risk areas and we would expect to see improved clearance rates in these herds. Use of this model at a finer scale is limited by a lack of data. Further studies at the interface of badger and cattle populations are needed to narrow down parameter estimates. In particular, spatially and temporally explicit badger prevalence data to match the detailed cattle data that are available would allow more detailed predictions to be made at a local scale.

## Supplementary Material

Additional calculations and derivations. Model code

## Data accessibility

The estimates used in this manuscript are from published literature. Estimates of testing during 2001 were made using VetNet, the UK national TB testing database and summary data are included in the electronic supplementary material. Requests for further extracts of veterinary surveillance data can be made to Defra at vetsurveillance@defra.gsi.gov.uk. Model code for reproducing the figures and results is included as a supplementary download.

## References

[1] Department for Environment Food and Rural Affairs (Defra). 2011 Bovine TB Eradication Programme for England.

[2] Murhead RH Burns KJ 1974 Tuberculosis in wild badgers in Gloucestershire: epidemiology. Vet. Rec. 95, 552–555. (10.1136/vr.95.24.552) 4462735

[3] Cheeseman CL Wilesmith JW Stuart FA 1989 Tuberculosis: the disease and its epidemiology in the badger, a review. Epidemiol. Infect. 103, 113–125. (10.1017/S0950268800030417) 2673822 PMC2249483

[4] Biek R 2012 Whole genome sequencing reveals local transmission patterns of *Mycobacterium bovis* in sympatric cattle and badger populations. PLoS Pathog. 8, e1003008 (10.1371/journal.ppat.1003008) 23209404 PMC3510252

[5] Goodchild AV Watkins GH Sayers AR Jones JR Clifton-Hadley RS 2012 Geographical association between the genotype of bovine tuberculosis in found dead badgers and in cattle herds. Vet. Rec. 170, 259 (10.1136/vr.100193) 22331501

[6] Bourne FJ The Independent Scientific Group on cattle TB 2007 bovine TB: the scientific evidence. Final report no. 4.

[7] Donnelly C Hone J 2010 Is there an association between levels of bovine tuberculosis in cattle herds and badgers? Stat. Commun. Infect. Dis. 2 (10.2202/1948-4690.1000)

[8] Donnelly CA Nouvellet P 2013 The contribution of badgers to confirmed tuberculosis in cattle in high-incidence areas in England. *PLoS Curr. Outbreaks*, 10 October 2013, edn 1 (10.1371/currents.outbreaks.097a904d3f3619db2fe78d24bc776098) PMC3992815 24761309

[9] Woodroffe R 2006 Culling and cattle controls influence tuberculosis risk for badgers. Proc. Natl Acad. Sci. USA 103, 14 713–14 717. (10.1073/pnas.0606251103) PMC1586183 17015843

[10] Smith GC 2001 Models of *Mycobacterium bovis* in wildlife and cattle. Tuberculosis 81, 51–64. (10.1054/tube.2000.0264) 11463224

[11] Mathews F 2006 Bovine tuberculosis (*Mycobacterium bovis*) in British farmland wildlife: the importance to agriculture. Proc. R. Soc. B 273, 357–365. (10.1098/rspb.2005.3298) PMC1560044 16543179

[12] Wilkinson D Smith GC Delahay RJ Cheeseman CL 2004 A model of bovine tuberculosis in the badger *Meles meles*: an evaluation of different vaccination strategies. J. Appl. Ecol. 41, 492–501. (10.1111/j.0021-8901.2004.00898.x)

[13] Delahay RJ Walker N Smith GC Smith GS Wilkinson D Clifton-Hadley RS Cheeseman CL Tomlinson AJ Chambers MA 2013 Long-term temporal trends and estimated transmission rates for *Mycobacterium bovis* infection in an undisturbed high-density badger (*Meles meles*) population. Epidemiol. Infect. 141, 1445–1456. (10.1017/S0950268813000721) 23537573 PMC9151602

[14] Cox DR Donnelly CA Bourne FJ Gettinby G McInerney JP Morrison WI Woodroffe R 2005 Simple model for tuberculosis in cattle and badgers. Proc. Natl Acad. Sci. USA 102, 17 588–17 593. (10.1073/pnas.0509003102) PMC1292989 16306260

[15] Conlan AJK McKinley TJ Karolemeas K Brooks-Pollock E Goodchild AV Mitchell AP Birch CPD Clifton-Hadley RS Wood JLN 2012 Estimating the hidden burden of bovine tuberculosis in Great Britain. PLoS Comput. Biol. 8, e1002730 (10.1371/journal.pcbi.1002730) 23093923 PMC3475695

[16] Brooks-Pollock E Conlan AJK Mitchell AP Blackwell R McKinley TJ Wood JLN 2013 Age-dependent patterns of bovine tuberculosis in cattle. Vet. Res. 44, 27 (10.1186/1297-9716-44-97) 24131703 PMC3853322

[17] Brooks-Pollock E Roberts GO Keeling MJ 2014 A dynamic model of bovine tuberculosis spread and control in Great Britain. Nature 511, 228–231. (10.1038/nature13529) 25008532

[18] Roberts MG Heesterbeek JAP 2003 A new method for estimating the effort required to control an infectious disease. Proc. R. Soc. B 270, 1359–1364. (10.1098/rspb.2003.2339) PMC1691377 12965026

[19] Heesterbeek JAP Roberts MG 2007 The type-reproduction number T in models for infectious disease control. Math. Biosci. 206, 3–10. (10.1016/j.mbs.2004.10.013) 16529777

[20] Garnett BT Delahay RJ Roper TJ 2002 Use of cattle farm resources by badgers (*Meles meles*) and risk of bovine tuberculosis (*Mycobacterium bovis*) transmission to cattle. Proc. R. Soc. B 269, 1487–1491. (10.1098/rspb.2002.2072) PMC1691052 12137579

[21] Wilkinson D Smith GC Delahay RJ Rogers LM Cheeseman CL Clifton-Hadley RS 2000 The effects of bovine tuberculosis (*Mycobacterium bovis*) on mortality in a badger (*Meles meles*) population in England. J. Zool. 250, 389–395. (10.1111/j.1469-7998.2000.tb00782.x)

