# Supplementary material for: Eliminating bovine tuberculosis in cattle and badgers: insight from a dynamic model
Source: Proc Biol Sci. 2015 Jun 7;282(1808):20150374. doi: 10.1098/rspb.2015.0374 (PMC4455805; doi:10.1098/rspb.2015.0374)

Supplementary Information for

Eliminating bovine tuberculosis in cattle and badgers: insight from a dynamic model

Ellen Brooks-Pollock[1*], James L.N. Wood[1]

[1]Disease Dynamics Unit, Department of Veterinary Medicine, University of Cambridge, Madingley Road, Cambridge, CB3 0ES, UK.

Table of Contents

Estimated reduction in cattle removal rate during the 2001 Foot and Mouth (FMD) epidemic. **1**

Equilibrium prevalence of infection **3**

Impact of cattle removal on badger infection prevalence **8**

Estimated reduction in cattle removal rate during the 2001 Foot and Mouth (FMD) epidemic.

The FMD outbreak began on 19 February 2001, after which testing for bovine TB in cattle was reduced or delayed (supplementary figure 1). However, because tests are generally scheduled during the winter months, the reduction in testing was not as severe as it might have been if the FMD outbreak had been earlier in the year. By the 19 February in the previous year, 20% of tests for that year had already taken place. Furthermore, not all tests were cancelled during the outbreak, in particular short interval tests (SIT) of herds identified as infected continued.

By comparing the number of tests that took place during weeks 1 to 7 of 2001 with the number of tests for the same period in the previous year, we estimated number of tests that would have taken place in the absence of FMD, assuming that testing patterns would have remained constant between the two years (sup table 1). Counting all test types, we estimate that 39% of usual tests took place during 2001.

Reactor detection rates vary considerably by test type, so we also used the average reactor rate by test type between 2000 and 2002 to estimate the number of reactors that would have been found if testing had not been reduced in 2001. As the majority of reactors are disclosed during short interval tests (SIT) and nearly 60% of SIT took place during 2001, we estimate that the removal rate of reactors was approximately 43% less due to the reduction in testing during FMD. This estimate provides a rough guide only, as the number of tests in a given year depends on the number of reactors found. Nevertheless, it provides a starting place for the sensitivity analysis in the main paper.

Supplementary Figure 1: The number of cattle tests by week and test type that took place in 2000 and 2001. The figure contains the most common test types: FT: follow-up 6 monthly and 12 monthly tests; CON: contiguous herd tests; RHT: routine herd tests; SIT: short interval tests; WHT: whole herd tests. Data extracted from the national bovine TB testing database, VetNet.

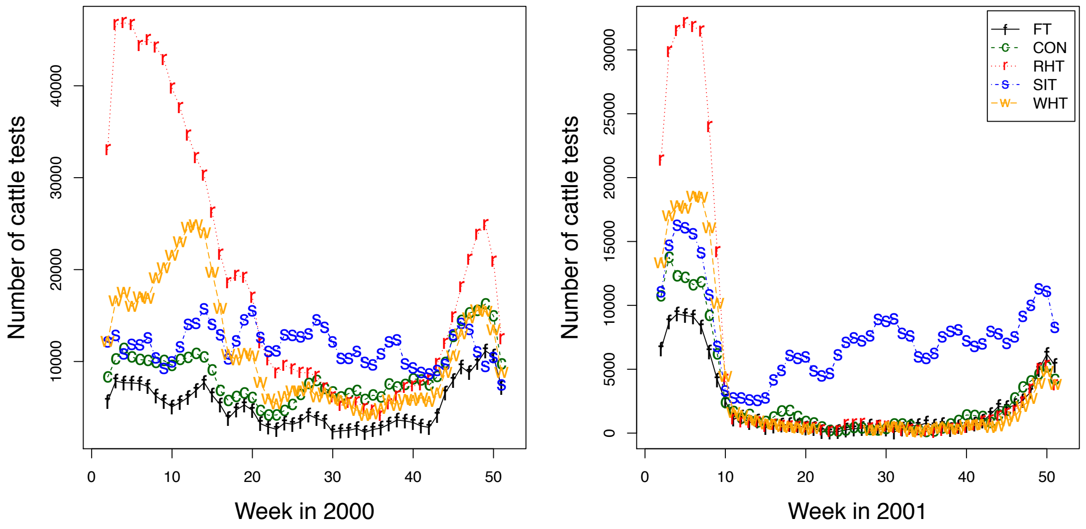

Supplementary table 1: Stages of the calculation to estimate the drop in reactor removal rate during 2001. The table contains the most common test types: 6M: 6 month follow up tests; 12M: 12 month follow-up tests; CON: contiguous herd tests; RHT: routine herd tests; SIT: short interval tests; WHT: whole herd tests. Data extracted from the national bovine TB testing database, VetNet.

**Test type**	**Average reactor rate (2000-2002)**	**# tests that would have taken place without FMD***	**# tests that did take place (% of total)**	**Estimated # reactors without FMD***	**# reactors with FMD**
6M	0.41%	192086	84242 (44%)	788	345
12M	0.41%	347555	33590 (10%)	1425	138
CON	0.38%	219072	88863 (41%)	832	338
CT	0.5%	73132	45015 (62%)	366	225
RHT	0.06%	670200	277947 (41%)	402	167
SIT	0.72%	689747	394599 (57%)	4966	2841
WHT	0.32%	624501	180439 (29%)	1998	577
**Total**		**2,816,293**	**1,104,695 (39%)**	**10777**	**4631 (57%)**

*Estimated by comparing testing patterns with the year 2000 (see text for details)

Equilibrium prevalence of infection

Following on from model equations (1) in the main text, to obtain the equilibria, we set each equation to zero. Taking the equation for I_C we have:

$$\frac{dI_C}{dt} = \beta_{CC} S_C I_C + \beta_{BC} S_C I_B - \gamma' I_C = 0$$

where $\gamma' = \gamma_C + \mu_C$ is the total removal rate of infected cattle due to testing and background slaughter rates. Setting $I_C = 1 - S_C$ and rearranging leads to a quadratic equation for S_C^* :

$$-\beta_{CC}\left(S_C^*\right)^2 + \left(\beta_{CC} + \beta_{BC} I_B + \gamma'\right) S_C^* - \gamma' = 0.$$

Therefore,

$$S_C^* = \frac{1}{2\beta_{CC}}\left(\beta_{CC} + \beta_{BC} I_B^* + \gamma' - \sqrt{\left(\beta_{CC} + \beta_{BC} I_B^* + \gamma'\right)^2 - 4\beta_{CC}\gamma'}\right).$$

The equivalent equation can be derived for S_B^* :

$$S_B^* = \frac{1}{2\beta_{BB}}\left(\beta_{BB} + \beta_{CB} I_C^* + \mu - \sqrt{\left(\beta_{BB} + \beta_{CB} I_C^* + \mu\right)^2 - 4\beta_{BB}\mu}\right).$$

where $\mu = \mu_B$. As there are only two infection states per host, these equations describe the system at equilibrium. We calculate the infection prevalence for a given set of parameters iteratively from a random starting point (say $I_B^* = 0.05$) until the solutions have stabilised.

These functional forms demonstrate how the equilibrium prevalence of infection depends on four variables: the within-host and between-host transmission rates, the prevalence of infection in the other host and the host-specific removal rate. To illustrate the relationships, supplementary figure 2 depicts the behaviour of I_C^* as a function of its four variables for a high transmission scenario of sustained transmission in cattle and badgers and intermediate inter-host transmission (scenario d in the main text). For a high transmission setting, the cattle removal rate has the biggest impact of cattle prevalence. It is notable that there are no threshold values for the prevalence of infection in badgers or the badger-to-cattle transmission rate; as spillover from badgers increases the fraction of infected cattle increases monotonically.

Supplementary Figure 2: The equilibrium prevalence of infected cattle as a function of four variables a) the cattle-to-cattle transmission rate; b) the badger-to-cattle transmission rate; c) the prevalence of infected badgers; and d) the removal rate of infected cattle.

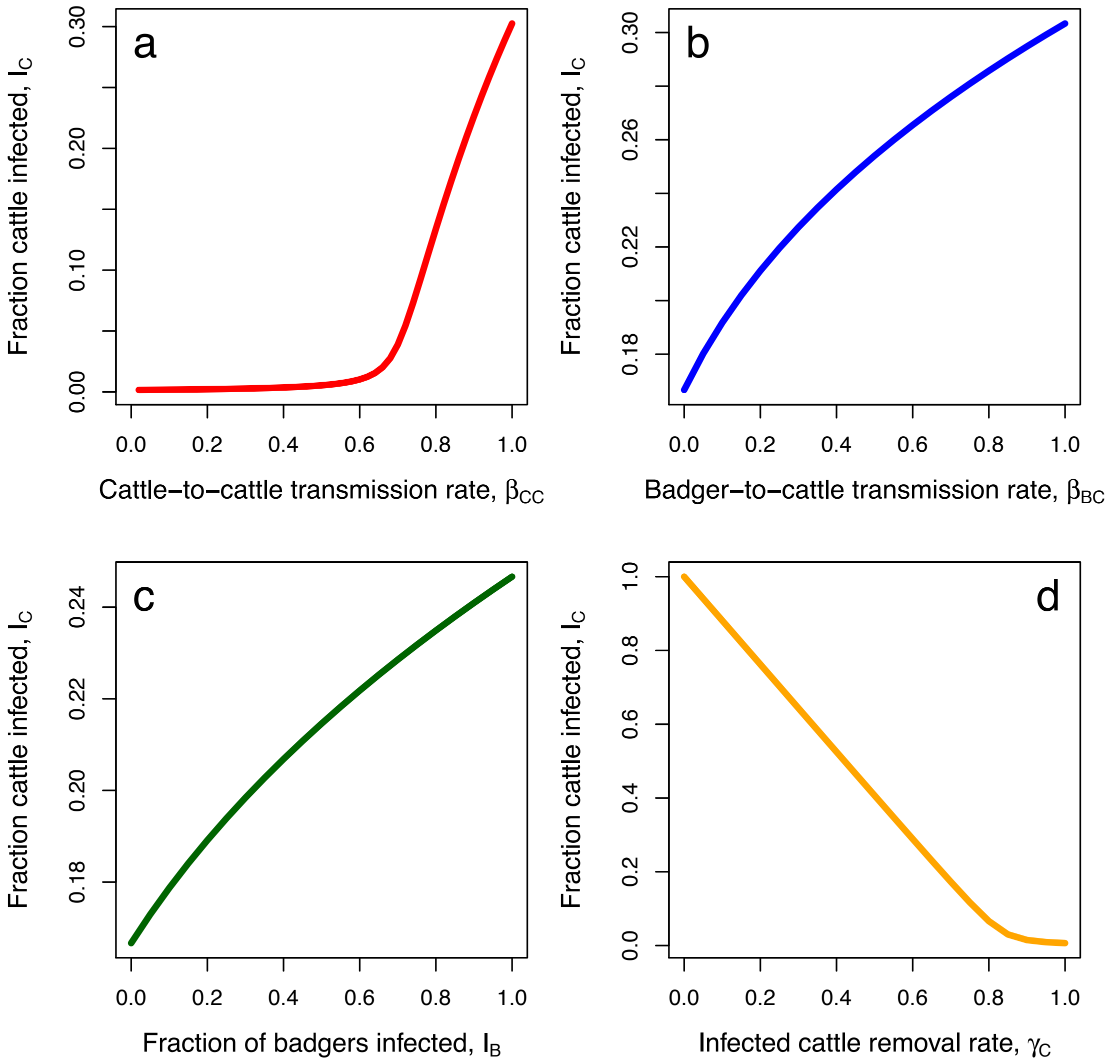

In the main paper we describe the impact of R_{CC} on infection prevalence in cattle and badgers (figure 1). Supplementary figures 3, 4 and 5 show the impact of R_{CB}, R_{BB} and R_{BC} for example high (dashed lines) and low (solid lines) incidence settings. In each figure, the blue lines indicate the proportion of badgers that are infected and the red lines the proportion of cattle. Supplementary figures 3 and 4 show that increasing cattle-to-badger transmission R_{CB} or badger-to-badger transmission R_{BB} has a modest impact on cattle infection prevalence, even under the high transmission scenario of high levels of inter-host and badger-to-badger transmission. As expected, increasing badger-to-cattle transmission R_{BC} is detrimental for the cattle population, even under the low transmission scenario (supplementary figure 4).

Supplementary Figure 3: Two scenarios for bovine tuberculosis transmission between cattle and badgers in Great Britain. The horizontal axis R_{CB} is the number of secondary cases in badgers

due directly to cattle and the vertical axis is the proportion of infected cattle (red) and infected badgers (blue) at equilibrium. R_{CC} R_{BC} , and R_{BB} are the number of secondary cases in cattle due to cattle, in cattle due to badgers and in badgers due to badgers. The two scenarios are a) low inter-species transmission and unsustained transmission in badgers; b) intermediate inter-species transmission and sustained transmission in badgers.

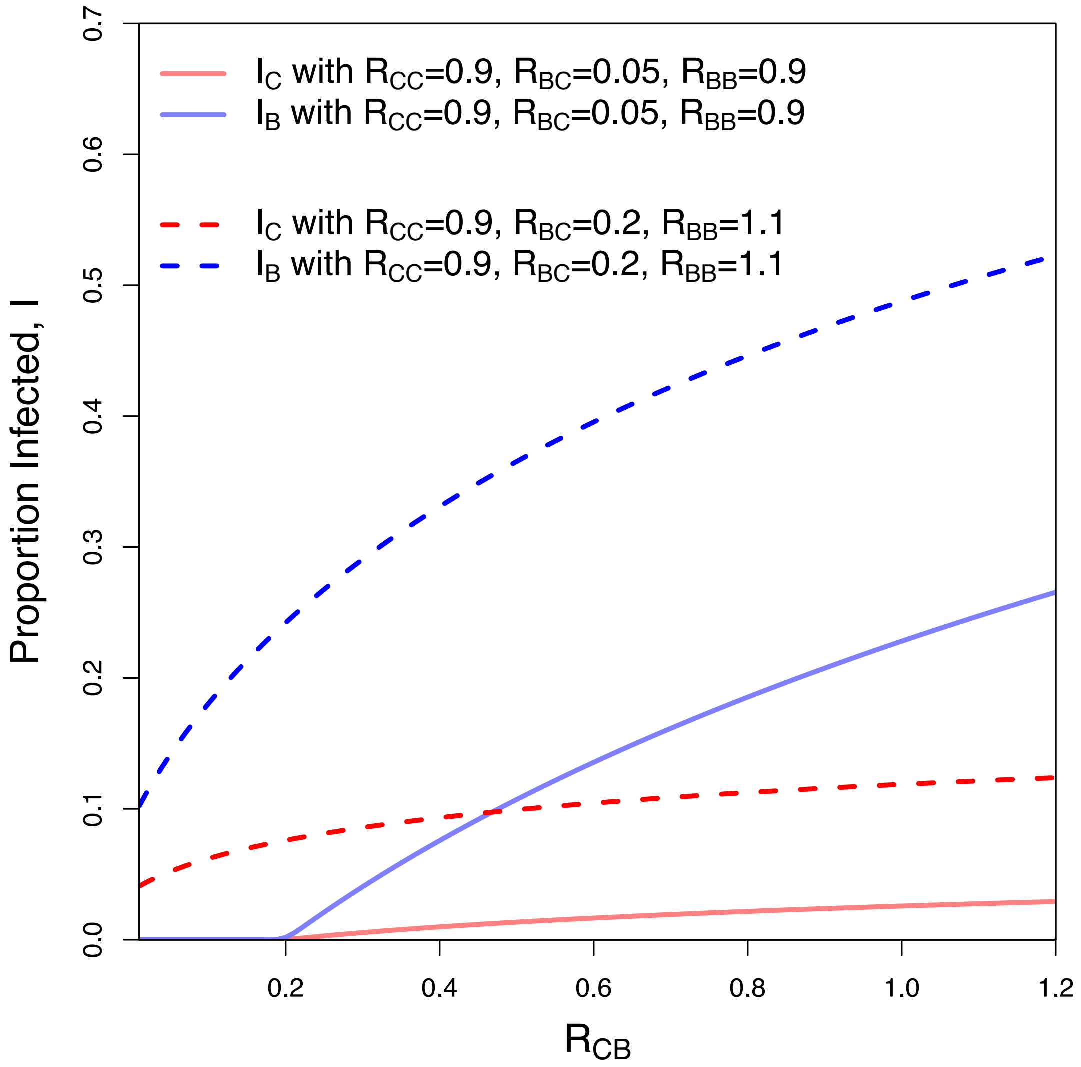

Supplementary Figure 4: Two scenarios for bovine tuberculosis transmission between cattle and badgers in Great Britain. The horizontal axis R_{BB} is the number of secondary cases in badgers due to badgers and the vertical axis is the proportion of infected cattle (red) and infected badgers (blue) at equilibrium. R_{CC} R_{BC} and R_{CB} are the number of secondary cases in cattle due to cattle, in cattle due to badgers and in badgers due to cattle. The two scenarios are a) low inter-species transmission from badgers to cattle and moderate transmission from cattle to badgers; b) intermediate inter-species transmission from badgers to cattle and high transmission from cattle to badgers.

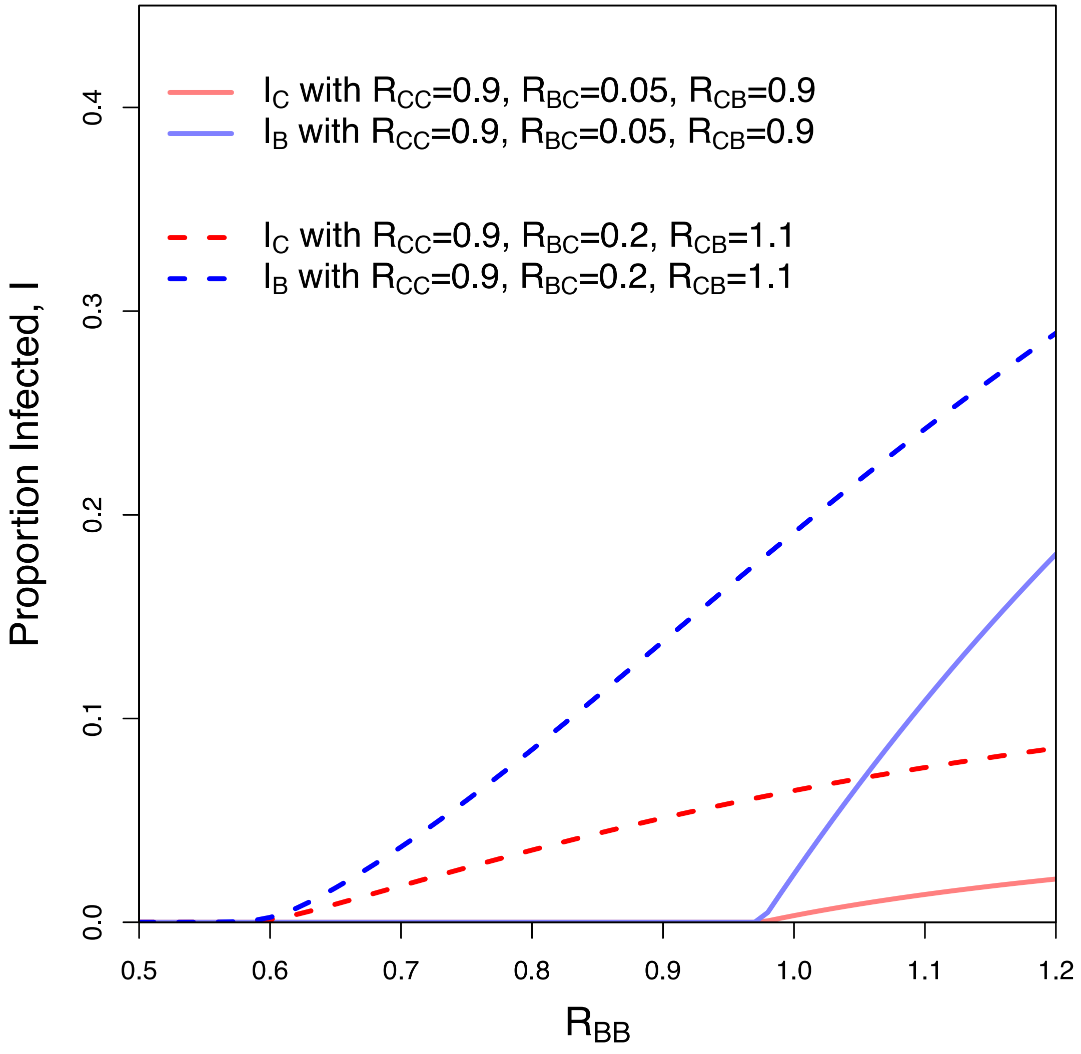

Supplementary Figure 5: Two scenarios for bovine tuberculosis transmission between cattle and badgers in Great Britain. The horizontal axis R_{BC} is the number of secondary cases in cattle due directly to badgers and the vertical axis is the proportion of infected cattle (red) and infected badgers (blue) at equilibrium. R_{CC} R_{CB} , and R_{BB} are the number of secondary cases in cattle due to cattle, in cattle due to badgers and in badgers due to badgers. The two scenarios are a) low inter-species transmission and unsustained transmission in badgers; b) intermediate inter-species transmission and sustained transmission in badgers.

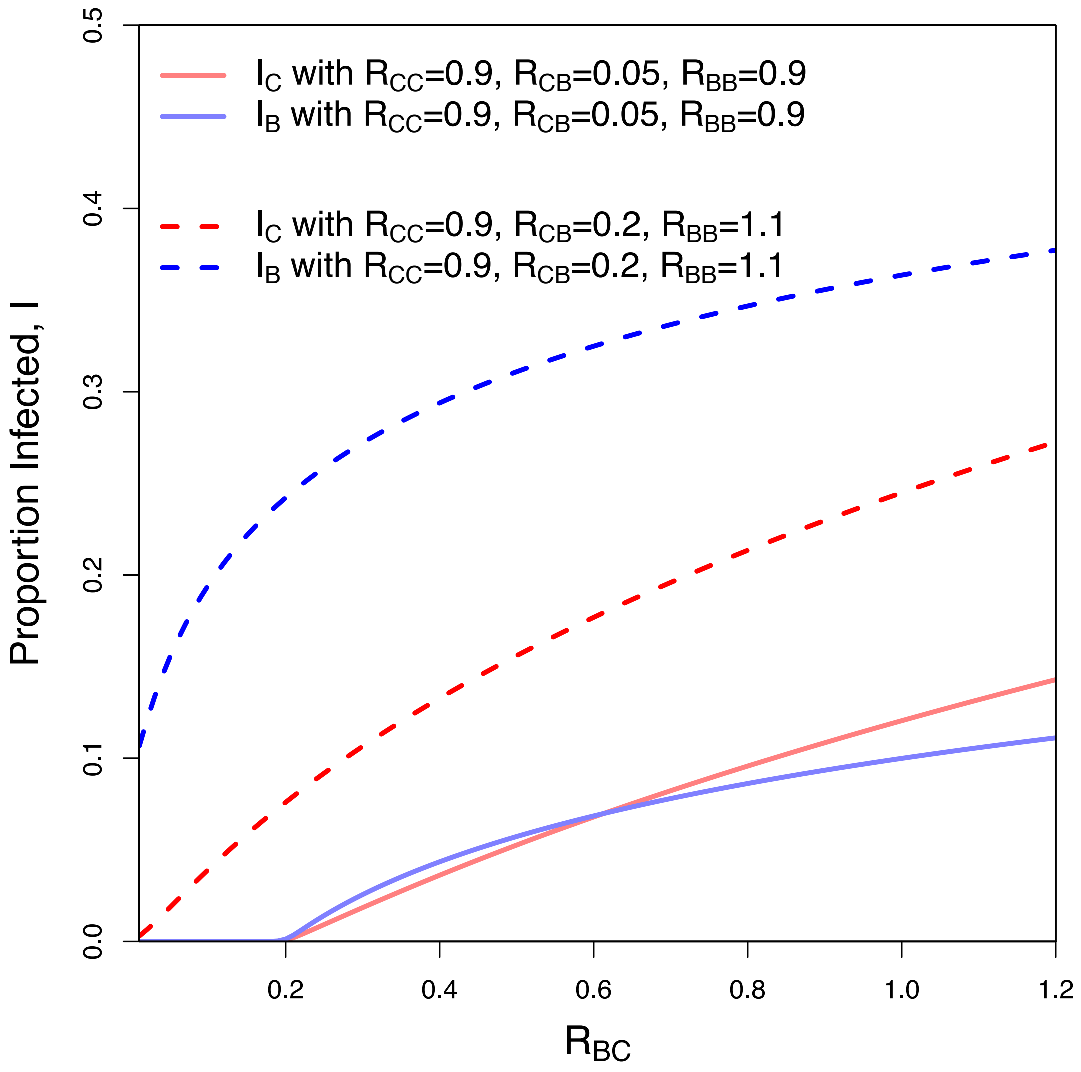

Impact of cattle removal on badger infection prevalence

Woodroffe et al. [9] observed an increase in badger infection prevalence when cattle testing was reduced. Figure 3 in the main paper illustrates the effect of reducing cattle testing on badger prevalence as a function of the badger-to-badger reproduction number. We additionally explored the impact of reducing cattle testing as a function of badger-to-badger reproduction number R_{BB} and cattle-to-badger reproduction number R_{CB}. Supplementary figure 6 shows the relative change in badger prevalence when cattle removal is reduced as a function of badger-to-badger reproduction number and the cattle-to-badger reproduction number. We assumed a 40% drop in cattle testing, inline with the estimates above and other parameters as in the main text ($R_{CC} = 1.05$ and $R_{BC} = 0.05$). As shown in figure 3 of the main text, we find that if $R_{BB} > 1.5$ cattle testing has almost no impact on infection prevalence in badgers. Supplementary figure 6 shows that for increasing values of R_{CB} the impact of reducing cattle testing is reduced, further supporting the argument that $R_{CB} \ll 0.2$.

Supplementary Figure 6: The relative change in badger prevalence when cattle testing is reduced as a function of badger-to-badger transmission R_{BB} and cattle-to-badger transmission R_{CB}.

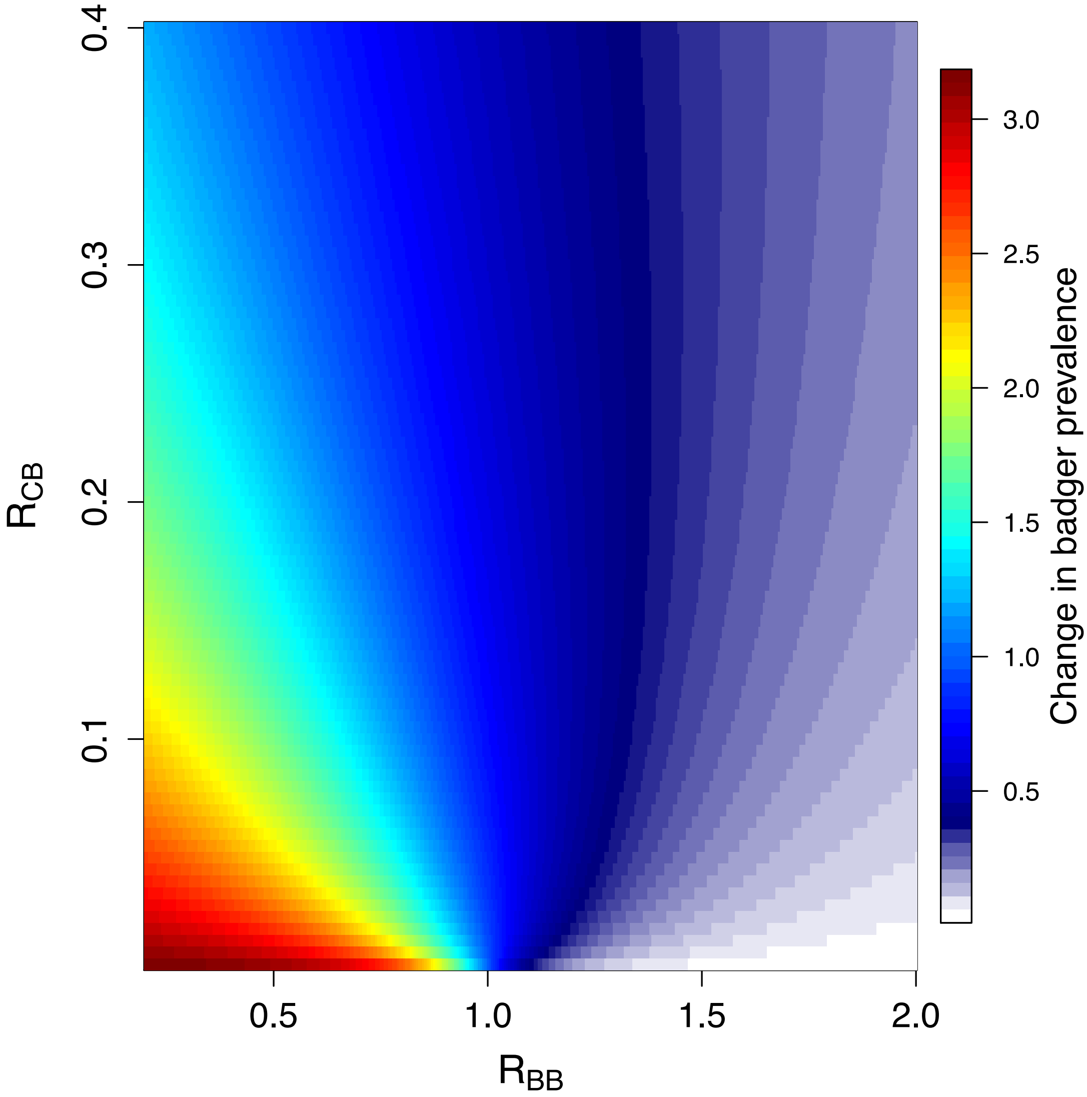

Supplement: Additional calculations and derivations. Model code [file rspb20150374supp1.pdf]
